# Hepatoprotective Effect of San-Cao Granule on Con A-Induced Liver Injury in Mice and Mechanisms of Action Exploration

**DOI:** 10.3389/fphar.2018.00624

**Published:** 2018-06-12

**Authors:** YuXue Yang, Ping Zhang, Yingying Wang, Shizhang Wei, Lu Zhang, Jiabo Wang, Xiaohua Lu, Houqin Zhou, Ruisheng Li, Jianxia Wen, Xuelin Zhou, Haotian Li, Kun Li, Yanling Zhao

**Affiliations:** ^1^College of Pharmacy, Chengdu University of Traditional Chinese Medicine, Chengdu, China; ^2^Department of Pharmacy, 302 Military Hospital of China, Beijing, China; ^3^Department of Integrative Medical Center, 302 Military Hospital of China, Beijing, China; ^4^Experimental Laboratory Center, 302 Military Hospital of China, Beijing, China

**Keywords:** San-Cao granule, hepatoprotective effect, active ingredients prediction, Con A-induced liver injury, anti-apoptosis

## Abstract

**Objective:** San-Cao granule (SCG), a traditional Chinese herb formula, has been used for treating autoimmune hepatitis (AIH) in our clinics for a long time. However, its active ingredients and mechanisms of action were still unknown due to its complicated chemical compositions. In the present study, the pharmacological study of SCG on acute liver injury induced by Concanavalin A (Con A) was performed to provide a scientific evidence for SCG against liver injury.

**Methods:** In order to screen active components and predicate mechanisms of action, an “ingredients-target-disease” interaction network was constructed by network pharmacology. Then, the pharmacological study was performed to evaluate the therapeutic effect and the underlying mechanisms of SCG on Con A-induced liver injury in mice.

**Results:** This research demonstrated the pharmacological effect of SCG on Con A-induced liver injury, which was through improving the liver function, relieving the pathological changes of liver tissue, decreasing the level of pro-inflammatory cytokines, and thus balancing the pro- and anti-inflammatory cytokines. And the anti-inflammatory of SCG may advantage over the ursodeoxycholic acid (UDCA). Network pharmacology analysis revealed that the pharmacological effect of SCG might be related to its active ingredients of taraxanthin, dihydrotanshinone I, isotanshinone I, γ-sitosterol, 3β-acetyl-20,25-epoxydammarane-24α, and δ-7-stigmastenol. The hepatoprotective effect of SCG was reflected by suppressing Con A-induced apoptosis which was mediated by TRAIL and FASL.

**Conclusion:** The combination of network pharmacology and experimental data has revealed the anti-apoptotic effect of SCG against Con A-induced liver injury.

## Introduction

Autoimmune hepatitis as a chronic progressive liver disease was characterized by immunological tolerance disorders targeting hepatocytes and inducing inflammation of liver parenchyma, eventually causing cirrhosis and liver failure (Krawitt, 2006; Manns and Vogel, 2006). Currently, for short of enough clinical samples from patients with AIH and the difficulties of manipulating in humans, the mechanism of AIH is hard to elucidate and there are few drugs for AIH. Up to now, diversified animal models for AIH study have been used to mimic human liver injury, and the induction reagents include Con A (Tiegs et al., 1992), Poly I:C (Dong et al., 2004), lipopolysaccharide (LPS; Yee et al., 2003), as well as alcohol consumption (Minagawa et al., 2004). Among them, Con A-induced immunological liver injury model, established by Tiegs et al. (1992) has been widely used for the resemblance of human AIH.

**FIGURE 1:**
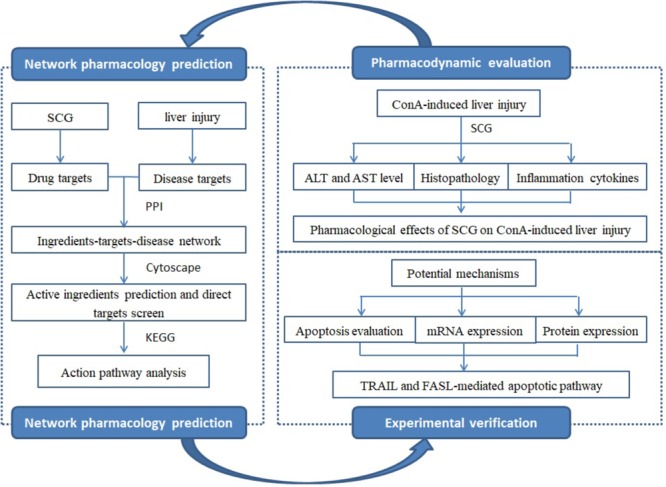
The framework of the present study.

The short- and long-term efficacy of immunosuppressive treatment, as the common used treatment, was controversial, and the severe adverse effect was demonstrated in a long-term use (Czaja, 2002). In recent years, TCMs were widely used in treating liver diseases with the safety and efficacy. SCG, developed by the TCMs clinicians, has been used for decades. It consists of *Rubia cordifolia* L., *Salvia miltiorrhiza* Bunge., *Siegesbeckia orientalis* L., *Forsythia suspensa* (Thunb.) Vahl., *Gentiana macrophylla* Pall., and *Glycyrrhiza uralensis* Fisch. at a weight ratio of 6:3:6:3:3:2. The clinical observation has demonstrated the hepatoprotective activity of SCG for AIH by improving liver function and alleviating the clinical symptoms (Zhang et al., 2011). In our previous study, time-of-flight liquid chromatography mass spectrometry mass spectrometer system (TOF LC/MS/MS) was used, and identified 15 chemical ingredients from SCG, including rubimaillin, purpurin, gentiopicroside, tanshinone IIA, salvianolic acid B, danshensu, glycyrrhizic acid, glycyrrhetinic acid, cryptotanshinone, alizarin, ferulic acid, formononetin, orientin, sweroside, and swertamarin (Wei et al., 2015). However, there were still limitations that the active ingredients of SCG and their targets were complicated, which caused us hard to illuminate its pharmacological mechanisms. Therefore, the current research aimed to explore the active ingredients of SCG and illustrate the underlying mechanism by combining network pharmacology and molecular biology technology (**Figure 1**).

## Materials and Methods

### Materials

Concanavalin A was purchased from Sigma (St Louis, MO, United States). ALT, AST, and MPO detection kits were purchased from Nanjing Jiancheng Bioengineering Institute (Nanjing, People’s Republic of China). Interleukin-2 (IL-2), IL-4, and IL-5 were measured by enzyme-linked immunosorbent assay (ELISA, eBioscience, San Diego, CA, United States). PrimerScript RT regent kit was supplied by Thermo Fisher Scientific (Waltham, MA, United States). ABI Step One Plus was purchased from Applied Biosystems Inc. (Carlsbad, CA, United States). Mouse anti-TRAIL, anti-IL-33, rabbit anti-DR5, anti-FASL, anti-FAS, and anti-caspase-3 primary antibodies were purchased from Abcam (United states, ab10515, ab54385, ab8416, ab15285, ab82419, ab13847) and anti-β-actin was purchased from ABclonal (China, AC026).

### Methods

#### SCG Ingredients and Related Targets Screening

In previous study, all ingredients from SCG were retrieved from the database of TCM Database @Taiwan (TDT^1^; Chen, 2011). The theoretical parameters, including oral bioavailability (OB) and drug-likeness (DL), of potential active ingredients were used for selection criteria (OB ≥ 30 and DL ≥ 0.18), as suggested by Traditional Chinese Medicine Systems Pharmacology (TCMSP) database^2^ (Liu et al., 2013). Herbal Ingredients’ Target (HIT) Database^3^ was used for the known ingredients targets screening (Ye et al., 2011). Through the structure similarity comparison, the putative targets were collected from Therapeutic Targets Database (TTD^4^; Chen et al., 2002).

#### Immune Hepatic Injury-Associated Treatment Targets Collection

Immune hepatic injury-associated gene and protein targets were screened from the database of Online Mendelian Inheritance in Man (OMIM^5^; Amberger et al., 2015). The Database of Interacting Proteins (DIP^6^) was also used for other interacting proteins of the above-mentioned targets (Yang et al., 2013). UniProt ID was the final ID type.

#### Network Construction and Analysis

To scientifically illuminate the relationships between SCG and immune hepatic injury, an “ingredients-target-disease” network was constructed by the protein–protein interaction (PPI) information, which was visualized and analyzed using Cytoscape v.3.5.1 (National Institute of General Medical Sciences, United States). Subsequently, CentiScaPe v.1.2 was used for analyzing the topological features (including degree, closeness centrality, and betweenness centrality), only with higher value of which were screened as the candidates for immune hepatic injury.

#### Herbal Preparation

The herb materials of SCG (including *R. cordifolia* L., *S. miltiorrhiza* Bunge., *S. orientalis* L., *F. suspensa* (Thunb.) Vahl., *G. macrophylla* Pall., and *G. uralensis* Fisch. at the weight ratio of 6:3:6:3:3:2) were obtained from Beijing Sheng Shi Long Herbs Co., Ltd. (Beijing, People’s Republic of China). The raw materials were extracted with 10-fold volume of water for twice by boiling 1 h for each time. The extract was merged and concentrated, and then added with ethanol slowly until the ratio of alcohol reached by 60%. After 24 h standing, the supernatant was collected and dried, and the dried material was mixed with moderate dextrin and pelletized with 95% ethanol. The yield of raw material was about 7.1%.

#### Animals and Experimental Design

Male Balb/c mice (6–8-weeks-old) were purchased from the animal center of Military Medical Sciences Academy of the PLA [Permission No SCXK-(A) 2016-0002]. According to the National Research Council Criteria, the animals were humane cared in accordance with the Chinese Animal Protection Act. The mice were kept in the pathogen-free conditions with temperature at 25 ± 0.5°C, humidity at 55 ± 5%, and light with 12:12 h light: dark cycle. The research was approved by Ethics Committee of 302 hospital of People’s Liberation Army.

In order to explore the toxic effect of SCG, we have conducted the acute toxicological test of SCG, and the result showed that there was no significant acute toxicity at the dose of 240 g/kg/day (maximum tolerance) for continuous 3 days.

To evaluate the treatment effect of SCG for Con A-induced liver injury, three doses [4.24, 2.12 (clinic equivalent dosage), and 1.06 g/kg/day] of SCG were orally administered to animals daily for 3 days before the Con A injection. Except for the normal group, Con A was dissolved in pyrogen-free PBS and administrated to mice by tail vein injection at a dosage of 20 mg/kg for liver injury induction, and the mice were sacrificed 12 h later according to the previous study (Heymann et al., 2015). Equal volume of saline was administrated to normal group. UDCA, as a positive control, was orally taken at a dose of 86.67 mg/kg/day.

#### Assessment of Liver Function

The serum levels of ALT and AST were used to assess the effect of SCG on liver function in Con A-treated mice. The MPO activity was measured to reflect the liver inflammation.

#### Histopathological Examination

For histopathology, liver sections of left lobe of all animals were collected and fixed in 4% paraformaldehyde buffer for 24 h, then dehydrated, embedded, and sectioned. Lastly, the sections were stained with hematoxylin and eosin (H&E).

#### Evaluation of Inflammatory Cytokines

The serum levels of inflammation cytokines, including IFN-γ and TNF-α, were measured by quantitative reverse transcription polymerase chain reaction (qRT-PCR). The primers are listed in **Table 1**. The serum levels of IL-2, IL-4, and IL-5 were measured by ELISA kits.

**Table 1:** Primer sequences used for RT-PCR.

Gene	Forward	Reverse
TRAIL	CCCTGCTTGCAGGTTAAGAG	GGCCTAAGGTCTTTCCATCC
FasL	GCAGCAGCCCATGAATTACC	AGATGAAGTGGCACTGCTGT CTAC
Caspase-3	GGTGGAGGCTGACTTCCTGT	GCGCGTACAGCTTCAGCAT
IL-33	ATGGGAAGAAGCTGATGGTG	CCGAGGACTTTTTGTGAAGG
Fas	CTCCGAGTTTAAAGCTGAGG	TGTACTCCTTCCCTTCTGTGC
IFN-γ	GTTACTGCCACGGCACAGTC	GCCAGTTCCTCCAGATATCCA
TNF-α	ACTGGCAGAAGAGGCACTCC	CTGCCACAAGCAGGAATGAG

#### Hepatocyte Apoptosis Examination

For the apoptosis detection, the paraffin-fixed liver sections were stained with the terminal deoxynucleotidyl transferase (TdT)-mediated dUTP nick end labeling (TUNEL) technique.

#### Quantitative RT-PCR Analysis

Quantitative RT-PCR was used to assess the effect of SCG on the mRNA expression of TRAIL, FASL, FAS, caspase-3, and IL-33 in the Con A-induced liver injury. Trizol reagent was used for the total RNA extraction from liver tissues. Subsequently, RNA (2 μg) was transcribed to cDNA using a PrimerScript RT regent kit. ABI Step One Plus was used for the subsequent PCR amplification. The 2^-ΔΔCT^ method was applied for the data analysis.

#### Western Blotting for the Protein Expression

To detect the effect of SCG on the protein expression of TRAIL, DR5 (the receptor of TRAIL), FAS, FASL, cleaved caspase-3, and IL-33, western blotting was performed. Liver tissues (100 mg) were homogenized and lysed in ice-cold lysis buffer with protease inhibitor. Then, the samples were centrifuged at 12,000 × *g* and 4°C for 10 min, and the supernatant was obtained for the subsequent western blotting with 12% SDS-PAGE. After transferred onto the polyvinylidene fluoride (PVDF) membrane, the blot was incubated in blocking buffer (TBST) with 5% non-fat milk powder for 2 h. Subsequently, immunodetection was performed using mouse anti-TRAIL (1:500), anti-IL-33 (1:1000), rabbit anti-DR5 (1:500), anti-FASL (1:1000), anti-FAS (1:1000), and anti-caspase-3 (1:1000) primary antibodies for 4 h, and anti-β-actin (1:10000) for 3 h, respectively. After 1-h incubation with respective secondary antibody, the membrane was washed in TBST for three times with 5-min interval for each time. Finally, the immunoreactivity bands were detected with chemiluminescence detection kit.

#### Statistical Analysis

Data were statistically analyzed using the SPSS Statistics 17.0 and presented as mean ±*SD*. One-way analysis of variance (ANOVA) was used for multiple groups’ comparison. A *P*-value lower than 0.05 was considered as statistically significant difference.

## Results

### Ingredients-Target Network Construction and Analysis

**FIGURE 2:**
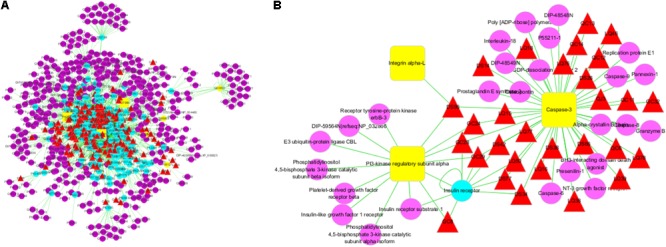
Ingredients-targets-disease interaction network of SCG. **(A)** The main network of SCG. **(B)** The sub-network with direct targets and the corresponding ingredients. The red triangles represent the potential active ingredients of SCG; the blue dot represents the indirect targets of SCG; the yellow dots stand for the targets of the special disease; and the purple dots represent the interaction targets of the specific disease and drugs.

First, we constructed an “ingredients-target-disease” network for presenting the noteworthy features with color-coded nodes for the effect of SCG on liver injury (**Figure 2A**). Among them, the yellow squares presented for the common targets of SCG and special disease and were the most noteworthy features for the further analysis. Subsequently, the common targets and the first neighbors of the network were segregated for screening active ingredients, showing the relationship of direct targets and the corresponding compounds (**Figure 2B**). The direct targets were integrin α-L, PI3K regulatory subunit α, and caspase-3. Insulin receptor, an indirect target, was correlated to the target of PI3K regulatory subunit α and caspase-3, which also made a big contribution.

### SCG Ingredients and Related Targets Screening

**FIGURE 3:**
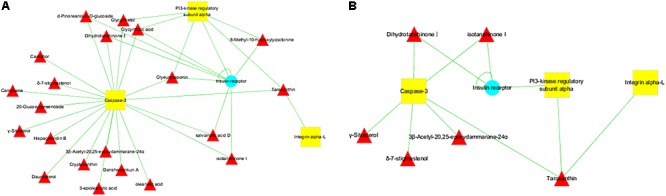
Potential active ingredients-targets interaction network of SCG. **(A)** The network of potential active components with related targets. **(B)** The segregated network of active components with related targets.

The active components with related targets were presented. As shown in **Figure 3A**, 21 potential active components were obtained, which included taraxanthin, glycyrrhizic acid, glyeurysaponin, glycyrrhetol, hispaglabridin B, 8-methyl-10-hydroxylycoctonine, dihydrotanshinone I, isotanshinone I, salvianolic acid D, oleanolic acid, γ-sitosterol, cryptoxanthin, danshenxinkun A, daucosterol, 20-glucosylginsenoside, 3β-acetyl-20,25-epoxydammarane-24α, camphene, camphor, δ-7-stigmastenol, D-pinoresinol-4-*o*-glucoside, and 3-epioleanolic acid. According to the TCMSP database criteria of OB ≥ 30% and DL ≥ 0.18, six ingredients, including taraxanthin, dihydrotanshinone I, isotanshinone I, γ-sitosterol, 3β-acetyl-20,25-epoxydammarane-24α, and δ-7-stigmastenol were collected for further investigation. Among them, taraxanthin might contribute the most for interacting all of the three direct targets (**Figure 3B** and **Table 2**).

**Table 2:** Information of active ingredients of SCG with the related targets and PK parameters.

Compound	Targets	Herbs	PK parameters	
			OB (%)	DL
Taraxanthin	PI3K regulatory subunit α; integrin α-L; caspase-3	*Salvia miltiorrhiza* Bunge.	38.3	0.55
Dihydrotanshinone I	Caspase-3; insulin receptor		45.04	0.36
Isotanshinone I			29.72	0.36
γ-Sitosterol	Caspase-3	*Salvia miltiorrhiza* Bunge.,	36.91	0.75
		*Glycyrrhiza uralensis* Fisch.		
3β-Acetyl-20,25-epoxydammarane-24α	Caspase-3	*Forsythia suspensa* (Thunb.) Vahl.	33.07	0.79
δ-7-stigmastenol			37.42	0.75

### Pathway Analysis Associated With SCG on Liver Injury

According to the above-mentioned results, three direct targets, including caspase-3, integrin α-L, and PI3K regulatory subunit α, were responsible for the efficacy of SCG on liver injury. However, the signal pathways participating in the pharmacological effect of SCG on liver injury are remaining unclear. After data retrieving from the KEGG database, the signaling pathways were put forwarded, as shown in **Table 3**. Among these pathways, the pathway of natural killer cell mediated cytotoxicity plays an important role for all three targets’ participation. The results indicated the anti-inflammatory and anti-apoptotic effects of SCG.

**Table 3:** Potential pathway of putative targets.

Term	Target count	*P*-value	Benjamin
Natural killer cell mediated cytotoxicity	Caspase-3, integrin α-L, PI3K regulatory subunit α	0.00031	0.027
Apoptosis	Caspase-3, PI3K regulatory subunit α	0.018	0.41
TNF signaling pathway	Caspase-3, PI3K regulatory subunit α	0.03	0.5
Leukocyte transendothelial migration	Caspase-3, PI3K regulatory subunit α	0.034	0.46
Rap1 signaling pathway	Caspase-3, PI3K regulatory subunit α	0.06	0.39
Regulation of actin cytoskeleton	Caspase-3, PI3K regulatory subunit α	0.06	0.37

### Pharmacological Effects of SCG on Liver Injury

**FIGURE 4:**
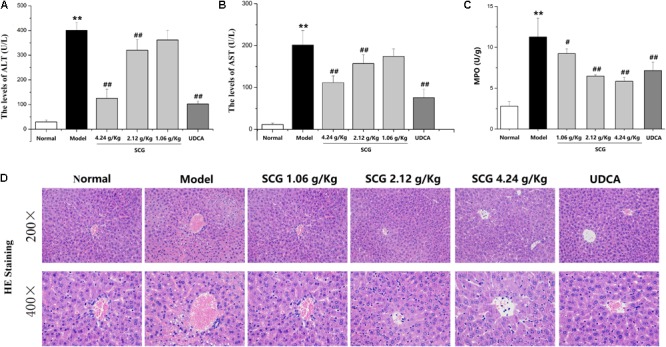
Pharmacological effect of SCG on Con A-induced liver injury in mice. **(A)** The serum level of ALT. **(B)** The serum level of AST. **(C)** The activity of MPO. **(D)** HE-stained liver section for histopathology evaluations and the histological changes. Data were expressed as mean ± *SD* (*n* = 6). ^∗∗^*P* < 0.01 compared with normal group; ^#^*P* < 0.05, ^##^*P* < 0.01 compared with model group.

As shown in **Figure 4**, administration of Con A significantly increased the serum levels of ALT and AST. Pre-administration of SCG at 4.24 and 2.12 g/kg, the levels of ALT and AST were remarkably decreased. Subsequently, HE staining was performed to assess the pathological changes of the liver tissues. The result showed that the liver tissues in mice of normal group were free of abnormal morphological changes and showed normal structures. The samples from Con A-treated mice showed severe inflammatory cell infiltration, cytoplasmic vacuolization, and massive hepatocyte apoptosis. Conversely, the extent of liver damage and the neutrophil infiltration were significantly diminished in SCG treatment groups, which was consistent with the result that SCG treatment could significantly inhibit the increase of MPO activity induced by Con A (**Figures 4C,D**). The effect of SCG was comparable to that of UDCA, which is used in clinical application against varies of hepatitis. These results indicated the remarkable pharmacological effects of SCG on Con A-induced liver injury by protecting hepatocytes from apoptosis and inflammatory infiltration.

### Effects of SCG on the Secretion of Inflammatory Cytokines

**FIGURE 5:**
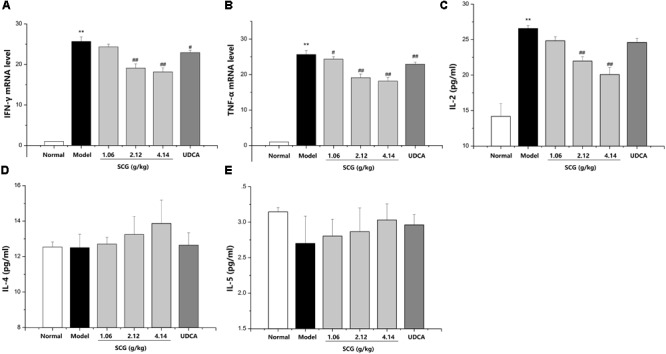
Effect of SCG on the levels of inflammatory cytokines in Con A-induced liver injury. **(A)** The expression level of IFN-γ. **(B)** The expression level of TNF-α. **(C)** The expression level of IL-2. **(D)** The expression of IL-4. **(E)** The expression level of IL-5. Data were expressed as mean ±*SD* (*n* = 6). ^∗∗^*P* < 0.01 compared with normal group; ^#^*P* < 0.05, ^##^*P* < 0.01 compared with model group.

Several cytokines, including inflammatory Th1 cytokines IFN-γ, TNF-α, IL-2, and anti-inflammatory Th2 cytokines IL-4 and IL-5, are critical molecular effectors in Con A-mediated acute hepatic injury (Gantner et al., 1995; Kusters et al., 1996; Abe et al., 2002; Santodomingo-Garzon et al., 2009). Administration of Con A induced a markedly increasing of IFN-γ, TNF-α, and IL-2, whereas there was no remarkable change on the levels of IL-4 and IL-5, which indicated the disorder of anti-inflammatory and pro-inflammatory. Furthermore, after oral administration of SCG in Con A-treated mice, the disorder was significantly suppressed by SCG through decreasing IFN-γ, TNF-α, and IL-2, and slightly increasing IL-4 and IL-5 (**Figure 5**).

### Effects of SCG on Hepatocyte Apoptosis

**FIGURE 6:**
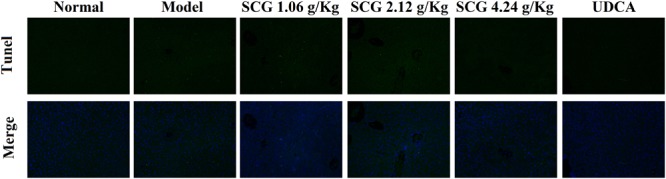
Effect of SCG on hepatocyte apoptosis in Con A-treated mice.

As indicated in the result of HE staining, pre-administration of SCG at 2.12 and 4.24 g/kg could significantly ameliorate the massive hepatocyte injury induced by Con A. In the sections, TUNEL staining was used to visually present the effect of SCG on hepatocyte apoptosis induced by Con A. As shown in **Figure 6**, Con A-treatment led to a vast apoptosis of hepatocyte. Contrastively, pretreatment of SCG could protect the hepatocyte from apoptosis with a dose-dependent manner, which indicated the anti-apoptotic activity of SCG.

### Effect of SCG on mRNA Expression of TRAIL, FASL, IL-33, and Caspase-3 in Con A-Treated Mice

**FIGURE 7:**
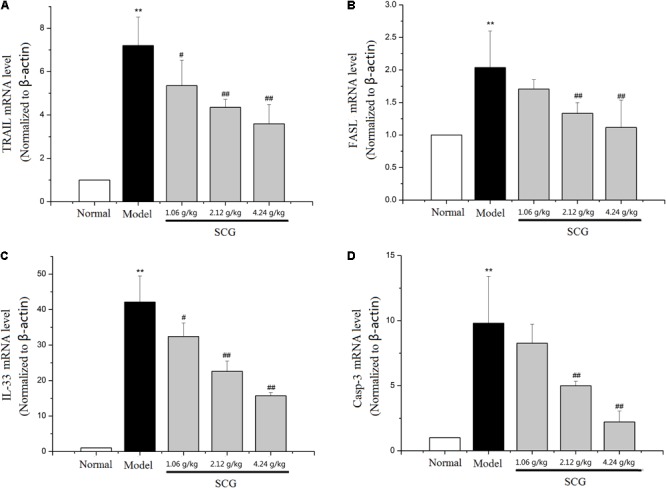
Effect of SCG on mRNA expression of TRAIL, FASL, IL-33, and caspase-3 in Con A-treated mice. **(A)** The mRNA level of TRAIL. **(B)** The mRNA level of FASL. **(C)** The mRNA level of IL-33. **(D)** The mRNA level of caspase-3. Data were expressed as mean ±*SD* (*n* = 6). ^∗∗^*P* < 0.01 compared with normal group; ^#^*P* < 0.05, ^##^*P* < 0.01 compared with model group.

It is reported that the cytotoxicity effector molecules and their receptors, such as FASL/FAS, TNF-α, and TRAIL/DR5, play critical roles in hepatocytes death during Con A-induced liver injury (Schumann and Tiegs, 1999; Kovalovich et al., 2001; Zheng et al., 2004). Through the pathway analysis of network pharmacology, the effect of SCG on apoptosis was found. In the present study, to clarify the mechanism of SCG on Con A-induced liver injury, the mRNA expression levels of TRAIL, FASL, IL-33, and caspase-3 were measured. As shown in **Figure 7**, Con A led to significant increasing of the mRNA expression of TRAIL and FASL. There was study showed that during Con A-induced liver injury, IL-33 was strongly expressed in damaged hepatocytes, which was mediated by FASL and TRAIL (Muhammad et al., 2012). Subsequently, we measured mRNA expression of IL-33 and caspase-3, and the result showed that the IL-33 and caspase-3 mRNA levels were significantly increased in model group, which indicated the occurrence of hepatocyte apoptosis. Conversely, in a dose-dependent manner, consecutively oral administration of SCG before the Con A-treatment significantly suppressed the increase of the mRNA expression of TRAIL, FASL, IL-33, and caspase-3 induced by Con A. These results indicated that SCG could inhibit Con A-induced hepatocyte apoptosis.

### Effect of SCG on Protein Expression of TRAIL, DR5, FASL, FAS, IL-33, and Cleaved Caspase-3 in Con A-Treated Mice

**FIGURE 8:**
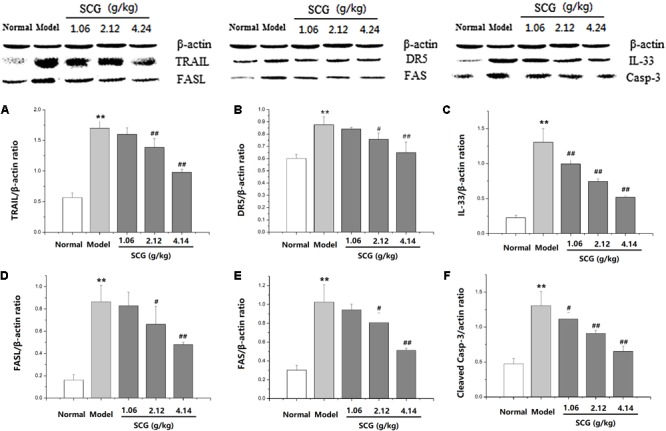
Effect of SCG on protein expression of TRAIL, DR5, FASL, FAS, IL-33, and caspase-3 in Con A-treated mice. **(A)** The expression of TRAIL. **(B)** The expression of DR5. **(C)** The expression of IL-33. **(D)** The expression of FASL. **(E)** The expression of FAS. **(F)** The expression of cleaved caspase-3. Data were expressed as mean ± *SD* (*n* = 3). ^∗∗^*P* < 0.01 compared with normal group; ^#^*P* < 0.05, ^##^*P* < 0.01 compared with model group.

Western blotting was performed to further explore the effect of SCG on the protein expression of TRAIL, DR5, FASL, FAS, IL-33, and cleaved caspase-3 (**Supplementary Data Sheet S2**). As shown in **Figure 8** and **Supplementary Data Sheet S1**, the expressions of TRAIL, DR5, FASL, FAS, IL-33, and cleaved caspase-3 were markedly increased in Con A-induced mice, when compared to the normal group. Furthermore, the administration of SCG significantly inhibited the expression of TRAIL, DR5, FASL, FAS, IL-33, and cleaved caspase-3. However, the effect is limited in response to the administration of SCG at 1.06 g/kg.

## Discussion

In recent years, more and more concentrations are focusing in network pharmacology during the research of TCMs, which can be used to analyze the relationship between drugs and diseases from proteome level (Sheng et al., 2014). It can present a hint to help us to understand the internal mechanism of TCM (Li et al., 2012).

In the present study, an integrated model of network pharmacology and experimental pharmacology was constructed to clarify the effect of SCG on Con A-induced acute liver injury, and simultaneously predict its potential active ingredients and mechanisms of action. For the first, network pharmacology was performed to explore the active ingredients and potential mechanisms of action. The result indicated that 21 potential active ingredients from *R. cordifolia* L., *S. miltiorrhiza* Bunge., *F. suspensa* (Thunb.) Vahl., *G. uralensis* Fisch., *G. macrophylla* Pall. were responsible to the pharmacological effect of SCG on liver injury. SCG as an oral preparation, the PK parameters of these compounds should be considered, in which OB and DL values are the most significant pharmacological characters. With consideration of OB and DL values, six active components, including taraxanthin, dihydrotanshinone I, isotanshinone I, γ-sitosterol, 3β-acetyl-20,25-epoxydammarane-24α, and δ-7-stigmastenol, were identified as the most valuable compounds for further research. Among them, taraxanthin from *S. miltiorrhiza* Bunge. contributed the most. Posteriorly, dihydrotanshinone I, and isotanshinone I in *S. miltiorrhiza* Bunge. also play an important role. There was also a hepatoprotective effect of γ-sitosterol, 3β-acetyl-20, 25-epoxydammarane-24α, and δ-7-stigmastenol by directly interacting with caspase-3.

After KEGG pathway enrichment and analysis, we found seven pathways participating in the pharmacological effect of SCG on immune-mediated liver injury, which include natural killer cell mediated cytotoxicity, apoptosis, TNF signaling pathway, leukocyte transendothelial migration, Rap1 signaling pathway, and regulation of actin cytoskeleton. All these pathways pointed out the anti-inflammatory and anti-apoptotic properties of SCG. Indeed, there could be a hint to explore the active ingredients and mechanisms of action.

Then, the pharmacological effect of SCG on Con A-induced liver injury was demonstrated. The results showed the pharmacological effect of SCG on Con A-induced liver injury by decreasing the serum level of ALT and AST, relieving the pathological changes of liver tissue, and balancing the pro and anti-inflammatory cytokines.

Concanavalin A-induced liver injury resembles the autoimmune or viral hepatitis in humans (Takeda et al., 2000). As reported, in the experimental model of Con A administration, the cytotoxicity effector molecules, and the receptors of them, such as perforin-granzyme, FASL/FAS, TNF-α, and TRAIL/DR5 in the pathway of natural killer cell mediated cytotoxicity play crucial roles for hepatitis development as well as hepatocyte cell death (Gantner et al., 1995; Watanabe et al., 1996; Kunstle et al., 1999; Zheng et al., 2004). On the other hand, the result of pathway analysis also indicated that the pathway of natural killer cell mediated cytotoxicity might contribute the most to the therapeutic effect of SCG on liver injury.

**FIGURE 9:**
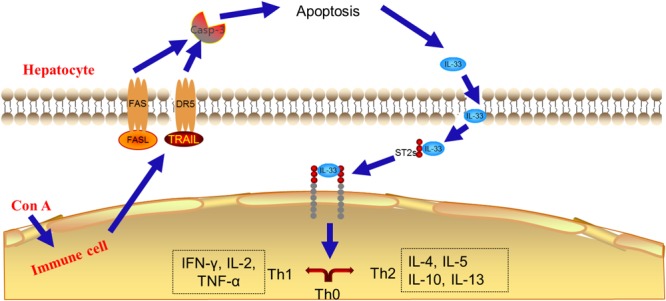
The summarized action pathway of SCG on Con A-induced liver injury.

Indeed, there were reports showed that the TRAIL^-/-^ or FASL^-/-^ mice or blockage of DR5 or FAS markedly suppressed the pathophysiology of Con A-mediated acute liver injury, suggesting the critical role of cytotoxic effectors released from immune cells in hepatitis development and hepatocyte cell death (Muhammad et al., 2012). IL-33, a member of IL-1 cytokines family, has been demonstrated to link to the progression of several liver diseases including acute, acute-to-chronic, and chronic hepatic injury. It is found that IL-33 may act as an “alarming mediator” during cell death associated with tissue damage (Lamkanfi and Dixit, 2009), and causes inflammatory cytokines secretion (Schmitz et al., 2005; Hayakawa et al., 2007). In the TRAIL/FASL-mediated apoptosis pathway, the cleaved form plays critical role (Luedde et al., 2014). The signal pathway was summarized, and is presented in **Figure 9**, which is consistent with the result of network pharmacology above that SCG could break TRAIL and FASL-mediated apoptosis pathway, thus balancing the pro- and anti-inflammatory cytokines.

Subsequently, molecular biology study was carried out to measure the effect of SCG on TRAIL- and FASL-mediated apoptosis pathway. The results presented that after the administration of Con A, the massive hepatocyte apoptosis was observed. The further exploration showed that the apoptosis might be caused by the high expression and secretion of TRAIL and FASL by immune cells, thus resulted in the high expression of DR5, FAS, IL-33, and cleaved caspase-3. Conversely, after the intervention of SCG to Con A-treated mice, the expression of TRAIL, DR5, FASL, FAS, IL-33, and cleaved caspase-3 was significantly decreased, indicating the anti-apoptosis effect of SCG on Con A-induced hepatic injury, which is consistent with the previous reports showing the immunosuppressive effect of *R. cordifolia* L. (Yang et al., 2000) *S. orientalis* L. (Shao et al., 2012), and *F. suspensa* (Thunb.) Vahl. (Zhang et al., 2015), and the immunological enhancement of *S. miltiorrhiza* Bunge. (Tang et al., 2011), and *G. uralensis* Fisch. (Wang et al., 2016).

## Conclusion

In summary, this study demonstrated the pharmacological effect of SCG on Con A-induced acute liver injury. The network pharmacology and experimental verification illustrated that SCG protected the liver against Con A in mice by suppressing TRAIL and FASL-mediated apoptosis pathway.

## Author Contributions

YY, PZ, YW, SW, and RL performed the experiments and wrote the manuscript. LZ, JbW, XL, and HZ analyzed the data. JxW, XZ, HL, and KL amended the paper. YZ designed the study and amended the paper.

## Conflict of Interest Statement

The authors declare that the research was conducted in the absence of any commercial or financial relationships that could be construed as a potential conflict of interest.

## Footnotes

1 http://tcm.cmu.edu.tw/

2 http://lsp.nwu.edu.cn/

3 http://lifecenter.sgst.cn/hit/

4 http://bidd.nus.edu.sg/group/cjttd/

5 http://www.omim.org/

6 http://dip.doe-mbi.ucla.edu

## Supplementary Material

DATA SHEET S1 The all original whole WB images.

DATA SHEET S2 The information of cleaved caspase-3.

## Abbreviations

AIH: autoimmune hepatitis
ALT: alanine transaminase
AST: aspartate transaminase
Con A: concanavalin A
DR5: death receptor 5
FASL: FAS ligand
IFN-γ: interferon-γ
IL-33: interleukin-33
MPO: myeloperoxidase
NR: no report
PI3K: phosphatidylinositol-3-kinase
PK: pharmacokinetic
SCG: San-Cao granule
TCMs: traditional Chinese medicines
TNF: tumor necrosis factor
TRAIL: tumor necrosis factor-related apoptosis inducing ligand
UDCA: ursodeoxycholic acid
