# Supplementary material for: Hepatoprotective Effect of San-Cao Granule on Con A-Induced Liver Injury in Mice and Mechanisms of Action Exploration
Source: Front Pharmacol. 2018 Jun 12;9:624. doi: 10.3389/fphar.2018.00624 (PMC6005824; doi:10.3389/fphar.2018.00624)
Supplement: DATA SHEET S1 — The all original whole WB images. [file Data_Sheet_1.DOCX]

**β-actin**

**
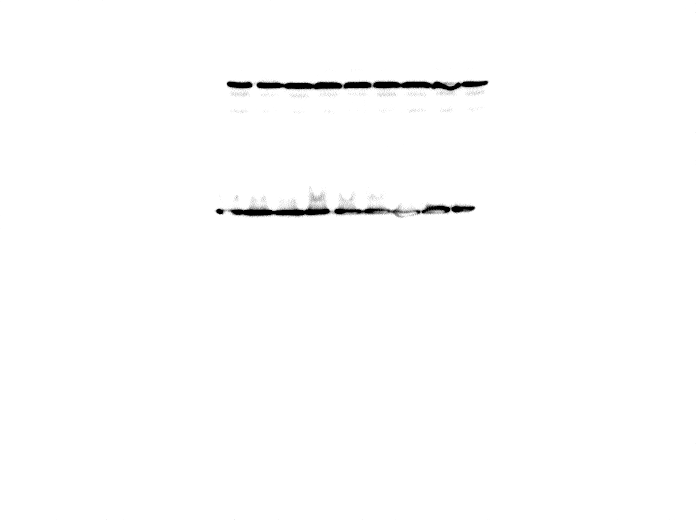
**

**
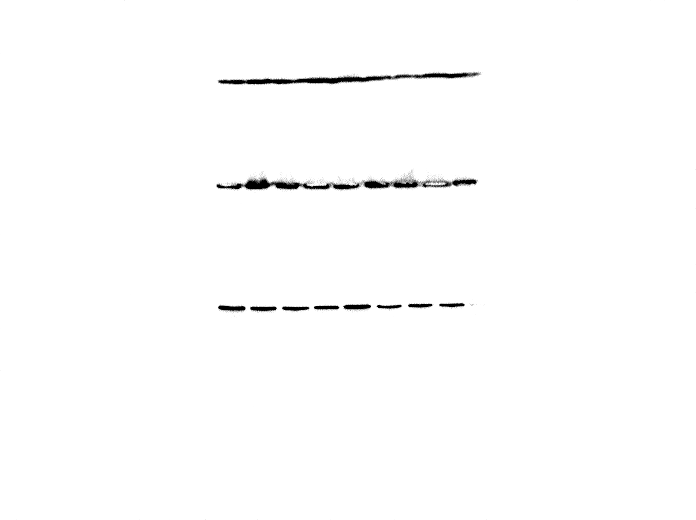
**

**
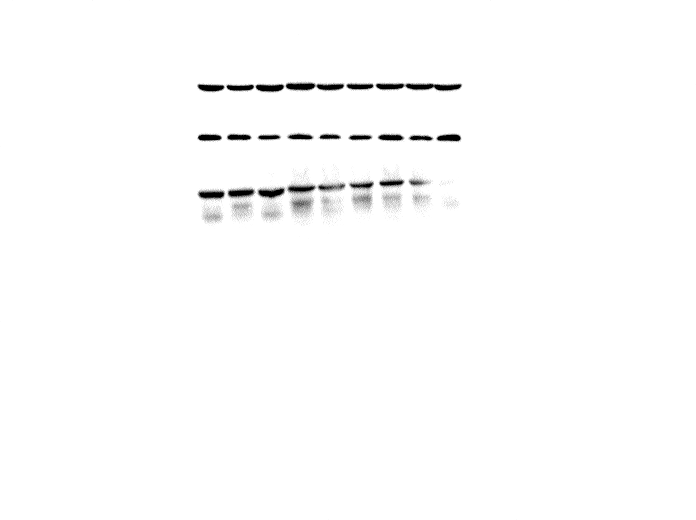
**

**Caspase-3**

**
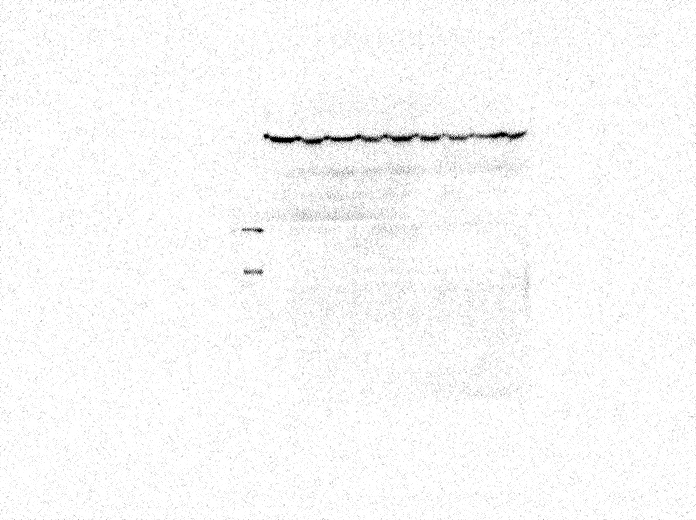
**

**
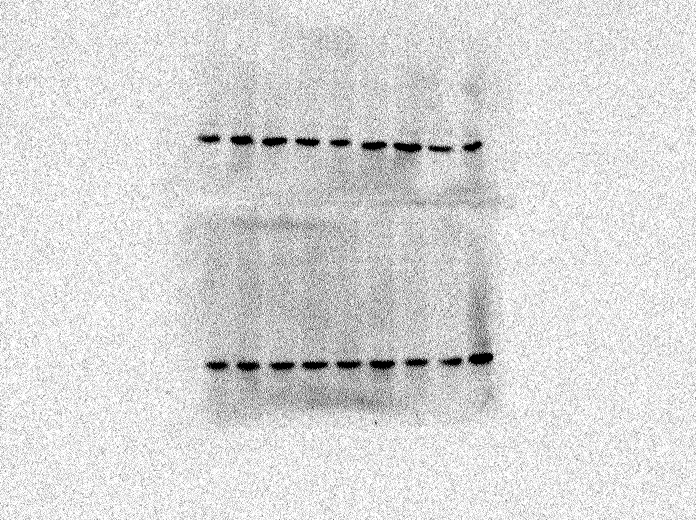
**

**
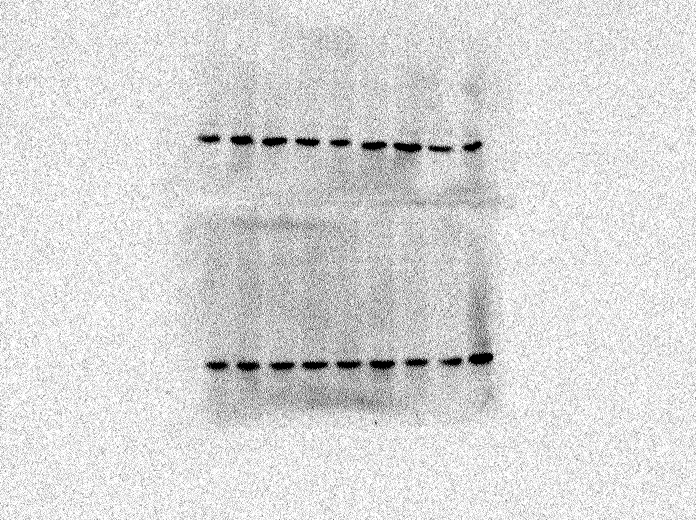
**

**DR5**

**The DR-5 antibody (ab8416) detects a band of approximately 60 kDa. In the present membrane, there were other antibody incubated on the membrane.**

**
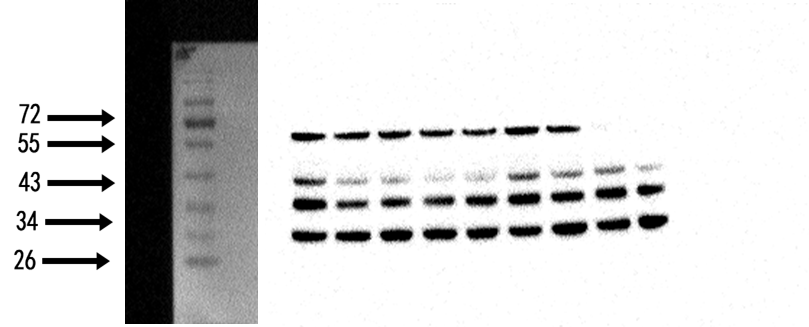
**

**
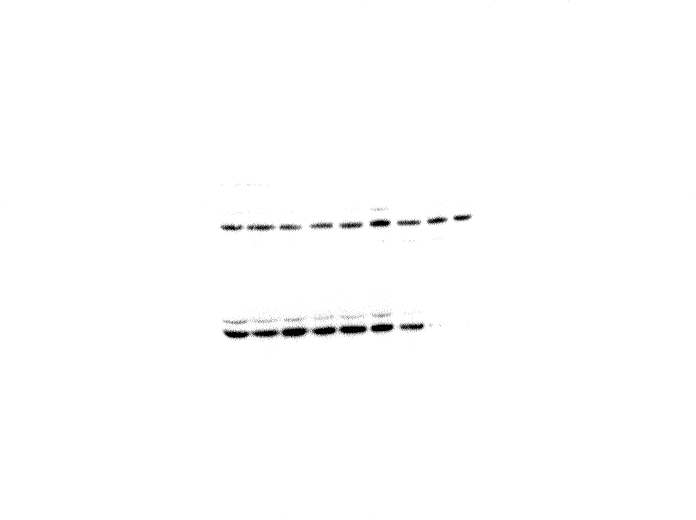
**

**
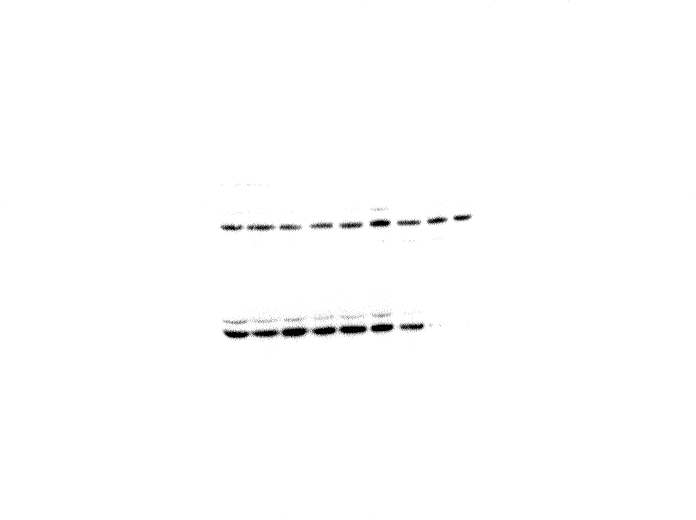
**

**FAS**

**
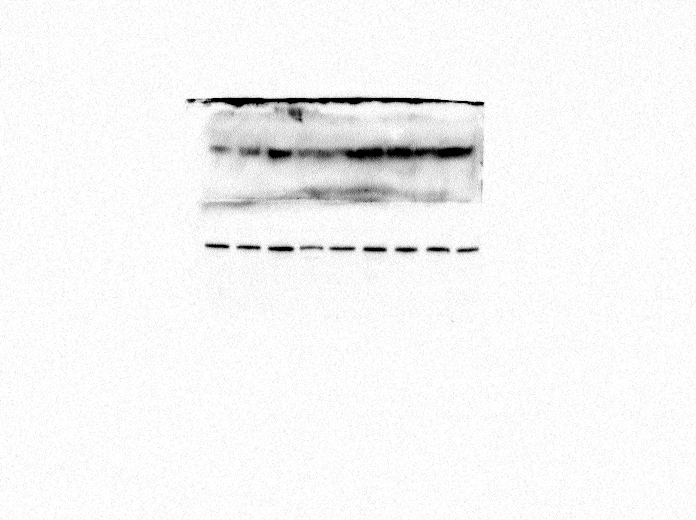
**

**
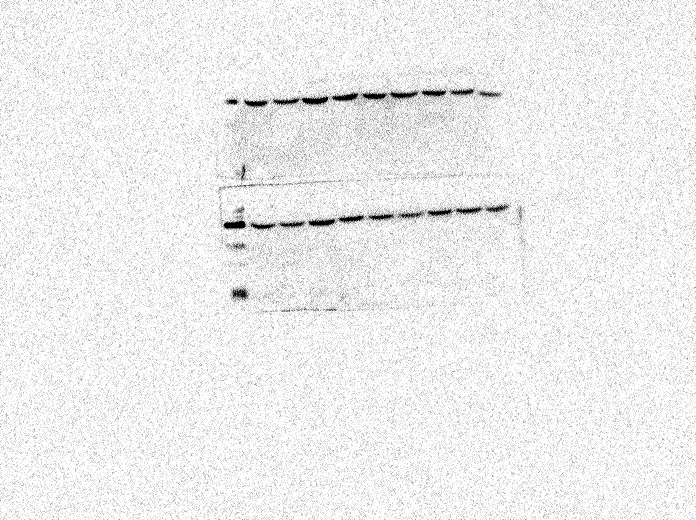
**

**
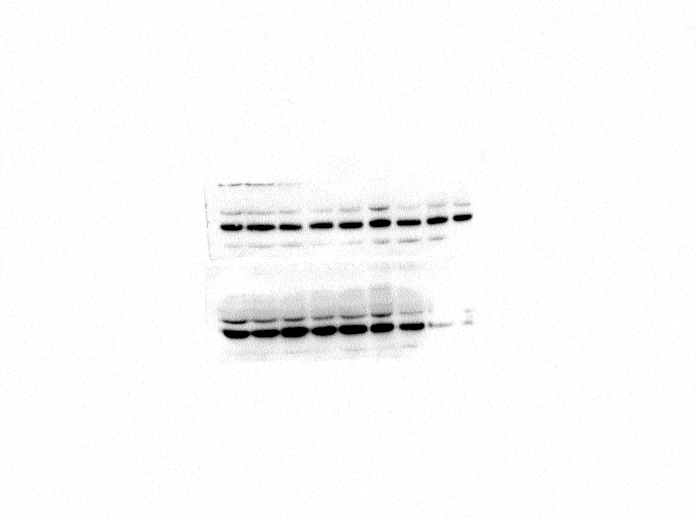
**

**FASL**

**
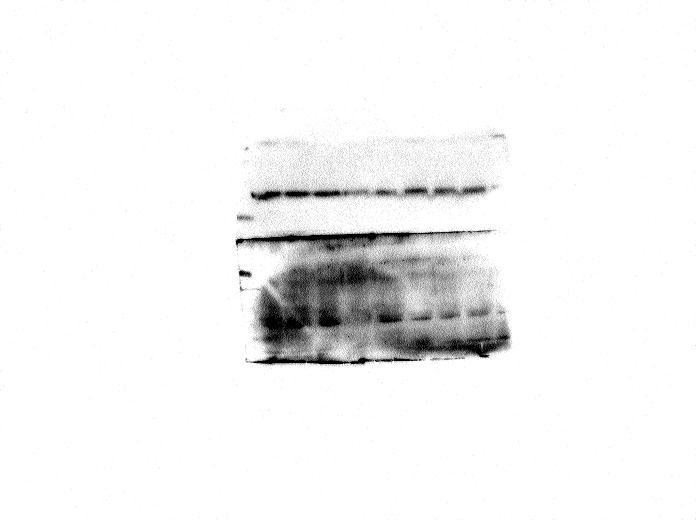
**

**
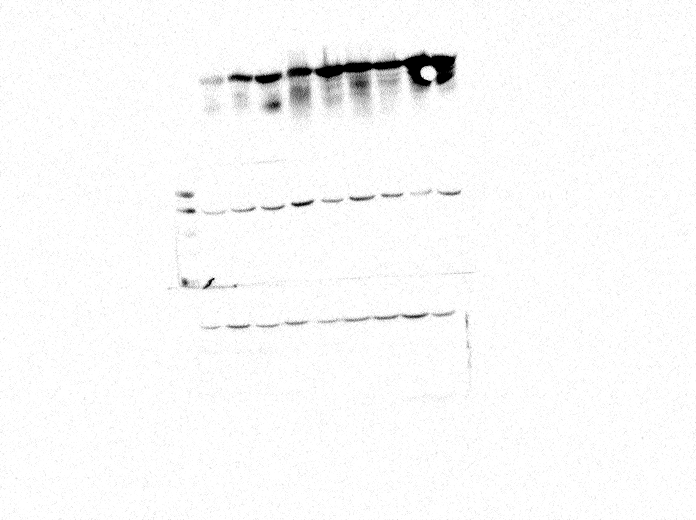
**

**
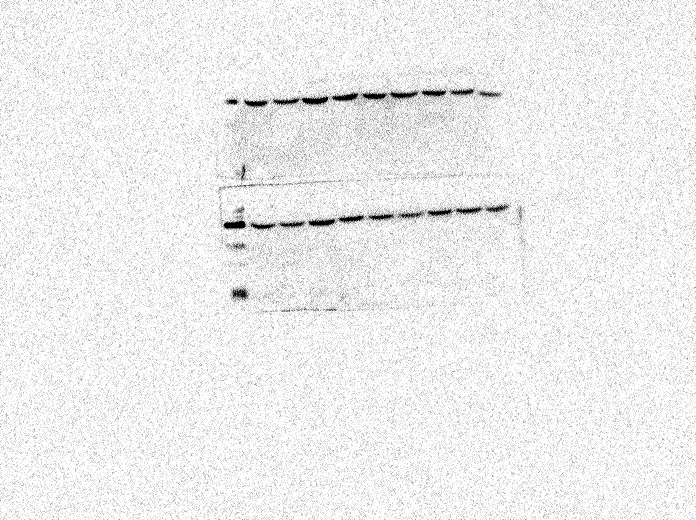
**

**Il-33**

**
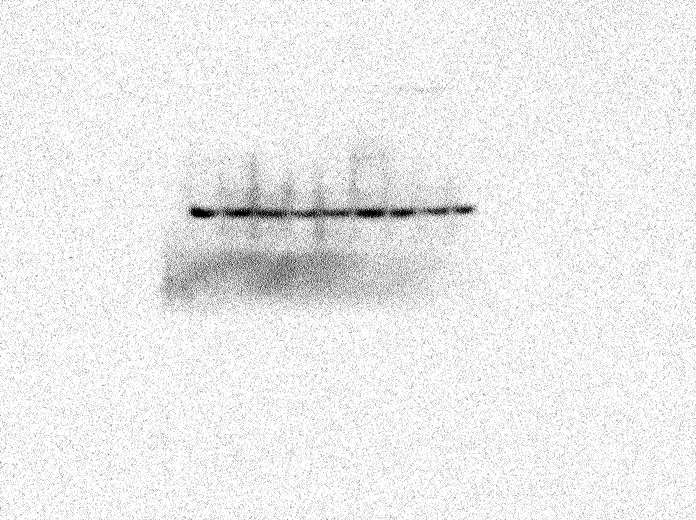
**

**
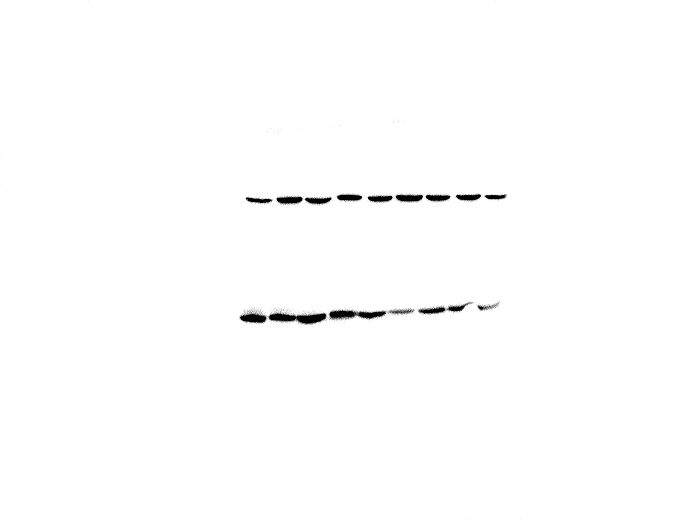

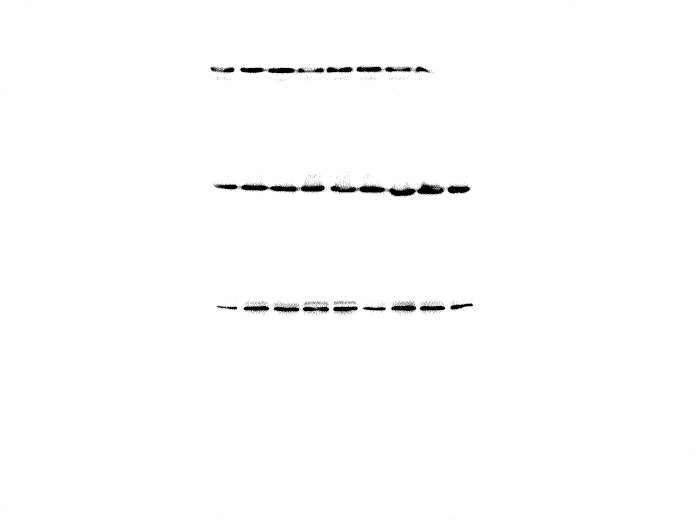
**

**Trail**

**
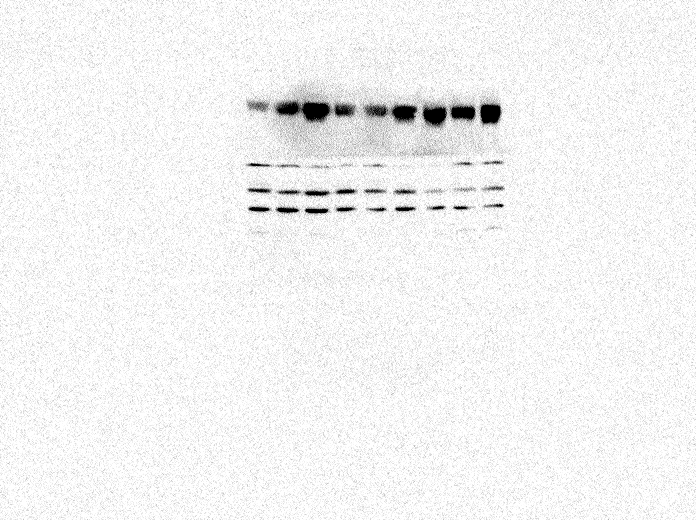
**

**
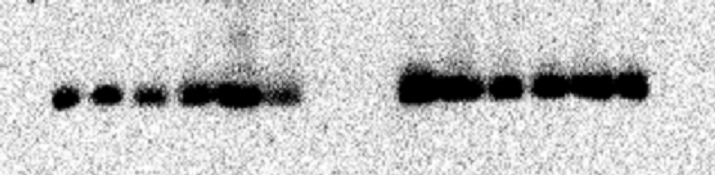
**
