# Supplementary material for: Hepatoprotective Effect of San-Cao Granule on Con A-Induced Liver Injury in Mice and Mechanisms of Action Exploration
Source: Front Pharmacol. 2018 Jun 12;9:624. doi: 10.3389/fphar.2018.00624 (PMC6005824; doi:10.3389/fphar.2018.00624)
Supplement: DATA SHEET S2 — The information of cleaved caspase-3. [file Data_Sheet_2.PDF]

abcam

Product datasheet

Anti-Caspase-3 antibody ab13847

KO VALIDATED

★★★★☆ 39 Abreviews 202 References 10 Images

Overview

**Product name**	Anti-Caspase-3 antibody
**Description**	Rabbit polyclonal to Caspase-3
**Host species**	Rabbit
**Specificity**	ab13847 recognizes a cleaved form of Caspase 3 (~17 kDa) after apoptosis has been induced in wildtype cells and not Caspase 3 knockout cells.
**Tested applications**	**Suitable for:** ICC/IF, IHC-P, IHC-Fr, WB, Flow Cyt, ICC, IHC (PFA fixed), IHC-FoFr, IHC - Wholemount
**Species reactivity**	**Reacts with:** Mouse, Rat, Human, Pig, Xenopus laevis, Drosophila melanogaster, Indian muntjac, Zebrafish, Rhesus monkey, Common marmoset, Schmidtea mediterranea, Salvelinus alpinus<br>**Predicted to work with:** Dog, Chinese hamster
**Immunogen**	Synthetic peptide corresponding to Human Caspase-3 aa 150-250 (internal sequence) conjugated to keyhole limpet haemocyanin.<br>(Peptide available as ab13848)
**Positive control**	This antibody gave a positive signal in WB with active Caspase 3 recombinant protein and pro-Caspase 3 recombinant protein. This antibody also gave a signal with HeLa staurosporine treated (2uM/4 hr) whole cell lysate.

Properties

**Form**	Liquid
**Storage instructions**	Shipped at 4°C. Store at +4°C short term (1-2 weeks). Upon delivery aliquot. Store at -20°C or -80°C. Avoid freeze / thaw cycle.
**Storage buffer**	pH: 7.40<br>Preservative: 0.02% Sodium azide<br>Constituent: PBS<br>Note: Batches of this product that have a concentration < 1mg/ml may have BSA added as a stabilising agent. If you would like information about the formulation of a specific lot, please contact our scientific support team who will be happy to help.
**Purity**	Immunogen affinity purified

**Clonality**	Polyclonal
**Isotype**	IgG

Applications

Our Abpromise guarantee covers the use of **ab13847** in the following tested applications.

The application notes include recommended starting dilutions; optimal dilutions/concentrations should be determined by the end user.

Application	Abreviews	Notes
ICC/IF	★★★★☆	Use a concentration of 5 µg/ml.
IHC-P	★★★★☆	1/50. Perform heat mediated antigen retrieval before commencing with IHC staining protocol.
IHC-Fr	★★★★★	Use at an assay dependent concentration.
WB	★★★★☆	1/500. Detects a band of approximately 17 kDa (predicted molecular weight: 17 kDa).
Flow Cyt	★★★★★	1/500.<br>ab171870 - Rabbit polyclonal IgG, is suitable for use as an isotype control with this antibody.
ICC	★★★★☆	Use at an assay dependent concentration.
IHC (PFA fixed)		1/300.
IHC-FoFr	★★★★★	1/300.
IHC - Wholemount	★★★★☆	1/500.

Target

**Function**	Involved in the activation cascade of caspases responsible for apoptosis execution. At the onset of apoptosis it proteolytically cleaves poly(ADP-ribose) polymerase (PARP) at a '216-Asp--Gly-217' bond. Cleaves and activates sterol regulatory element binding proteins (SREBPs) between the basic helix-loop-helix leucine zipper domain and the membrane attachment domain. Cleaves and activates caspase-6, -7 and -9. Involved in the cleavage of huntingtin.
**Tissue specificity**	Highly expressed in lung, spleen, heart, liver and kidney. Moderate levels in brain and skeletal muscle, and low in testis. Also found in many cell lines, highest expression in cells of the immune system.
**Sequence similarities**	Belongs to the peptidase C14A family.
**Post-translational modifications**	Cleavage by granzyme B, caspase-6, caspase-8 and caspase-10 generates the two active subunits. Additional processing of the propeptides is likely due to the autocatalytic activity of the activated protease. Active heterodimers between the small subunit of caspase-7 protease and the large subunit of caspase-3 also occur and vice versa.<br>S-nitrosylated on its catalytic site cysteine in unstimulated human cell lines and denitrosylated upon activation of the Fas apoptotic pathway, associated with an increase in intracellular caspase activity. Fas therefore activates caspase-3 not only by inducing the cleavage of the caspase zymogen to its active subunits, but also by stimulating the denitrosylation of its active site thiol.

Cellular localization Cytoplasm.

Images

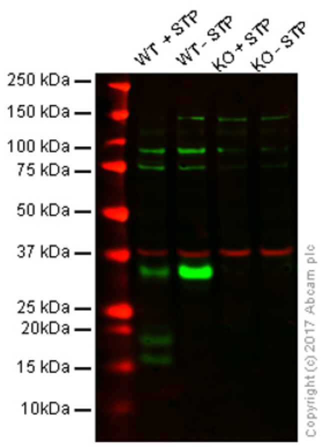

Western blot - Anti-Caspase-3 antibody (ab13847)

Lane 1: Wild-type HAP1 cell lysate + Staurosporine (ab146588) (1µM for 4h)
Lane 2: Wild-type HAP1 cell lysate
Lane 3: Caspase-3 knockout HAP1 cell lysate + Staurosporine (ab146588) (1µM for 4h)
Lane 4: Caspase-3 knockout HAP1 cell lysate

Lanes 1 - 4: Merged signal (red and green). Green - ab13847 observed at 17 kDa. Red - loading control, ab8245, observed at 37 kDa.

ab13847 was shown to recognise Caspase 3 when Caspase 3 knockout samples were used, along with additional cross-reactive bands. Wild-type and Caspase 3 knockout samples (±staurosporine treatment) were subjected to SDS-PAGE. ab13847 and ab8245 (loading control to GAPDH) were diluted to 1/500 and 1/10000 respectively and incubated overnight at 4°C. Blots were developed with Goat anti-Rabbit IgG H&L (IRDye® 800CW) preadsorbed (ab216773) and Goat anti-Mouse IgG H&L (IRDye® 680RD) preadsorbed (ab216776) secondary antibodies at 1/10000 dilution for 1 hour at room temperature before imaging.

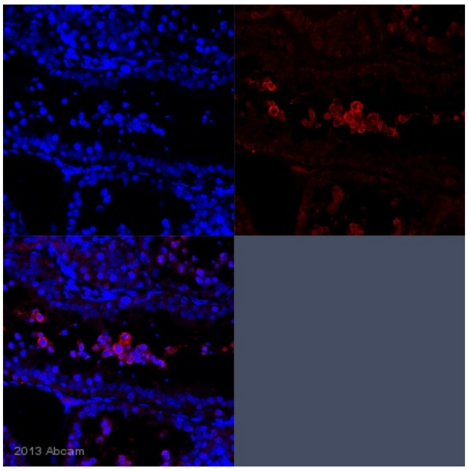

Immunohistochemistry (Formalin/PFA-fixed paraffin-embedded sections) - Anti-Caspase-3 antibody (ab13847)

This image is courtesy of an abreview from Zachary Yu-Ching Lin

IHC-P image of Caspase 3 staining with ab13847 on tissue sections from juvenile marmoset testis. The sections were subjected to heat-mediated antigen retrieval using Dako antigen retrieval solution. The sections were then blocked with 5% milk for 30 minutes at 25°C, before incubation with ab13847 (1/100 dilution) for 18 hours at 4°C. The secondary was an Alexa-Fluor 555 conjugated goat anti-rabbit polyclonal, used at a 1/500 dilution.

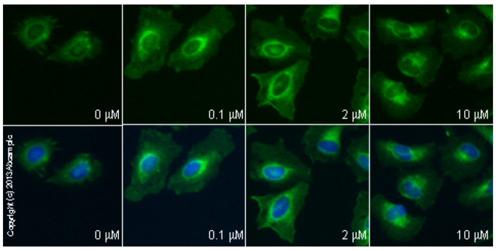

Immunocytochemistry/ Immunofluorescence - Anti-Caspase-3 antibody (ab13847)

ab13847 staining caspase 3 in HeLa cells treated with RTIL-13™ (ab120465), by ICC/IF. Increase in caspase 3 expression correlates with increased concentration of RTIL-13™, as described in literature.

The cells were incubated at 37°C for 24h in media containing different concentrations of ab120465 (RTIL-13™) in DMSO, fixed with 4% formaldehyde for 10 minutes at room temperature and blocked with PBS containing 10% goat serum, 0.3 M glycine, 1% BSA and 0.1% tween for 2h at room temperature. Staining of the treated cells with ab13847 (10 µg/ml) was performed overnight at 4°C in PBS containing 1% BSA and 0.1% tween. Goat Anti-Rabbit IgG H&L (DyLight® 488) preadsorbed (ab96899) at 1/250 dilution was used as the secondary antibody. Nuclei were counterstained with DAPI and are shown in blue.

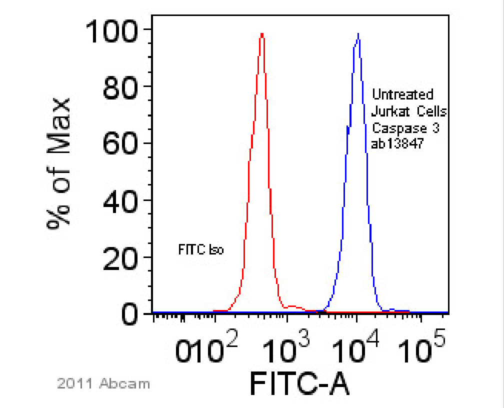

Flow Cytometry - Anti-Caspase-3 antibody (ab13847)

This image is courtesy of an anonymous Abreview

ab13847 staining active caspase 3 in Human Jurkat cells by Flow Cytometry. Cells were prepared in a phosphate buffered solution containing 0.1% sodium azide with FBS fixed with paraformaldehyde and permeabilized with Triton X-100 and NP40. The sample was incubated with the primary antibody (1/100 in wash buffer) for 24 hours at 4°C. A FITC-conjugated Goat anti-rabbit Ig (1/100) was used as the secondary antibody.

Gating Strategy: Isolate cell population from plot of SSC-A / FSA-A

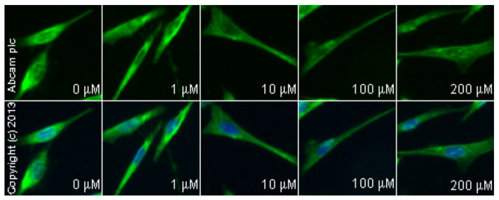

Immunocytochemistry/ Immunofluorescence - Anti-Caspase-3 antibody (ab13847)

ab13847 staining caspase 3 in SKNSH cells treated with Z-IETD-FMK (ab141382), by ICC/IF. Decrease in caspase 3 expression correlates with increased concentration of Z-IETD-FMK, as described in literature.
The cells were incubated at 37°C for 1 hour in media containing different concentrations of ab141382 (Z-IETD-FMK) in DMSO. After this incubation, 10 µM of camptothecin (ab120115) was added to all samples and the cells were incubated for further 24 hours. The samples were then fixed with 4% formaldehyde for 10 minutes at room temperature and blocked with PBS containing 10% goat serum, 0.3 M glycine, 1% BSA and 0.1% tween for 2h at room temperature. Staining of the treated cells with ab13847 (5 µg/ml) was performed overnight at 4°C in PBS containing 1% BSA and 0.1% tween. Goat Anti-Rabbit IgG H&L (DyLight® 488) preadsorbed (ab96899) at 1/250 dilution was used as the secondary antibody. Nuclei were counterstained with DAPI and are shown in blue.

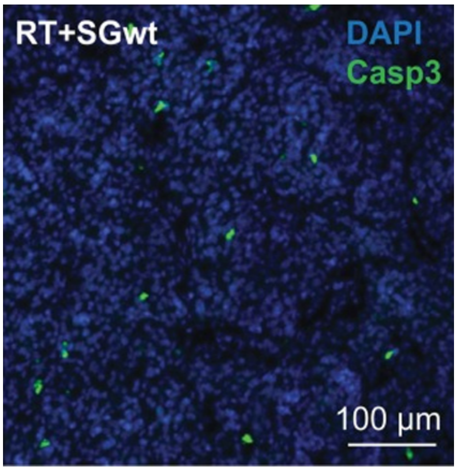

Immunohistochemistry (Frozen sections) - Anti-Caspase-3 antibody (ab13847)

Image from PLoS One. 2015; 10(5): e0126688. Fig 2b, doi: 10.1371/journal.pone.0126688

5µm frozen sections of tumor tissue were fixed with 100% ice cold methanol for 10 minutes, then blocked in 5% normal goat serum in PBS (pH 7.4) for 1h. Sections were incubated with ab13847 (1:500) at 4°C overnight and for 1h with secondary antibodies at room temperature.

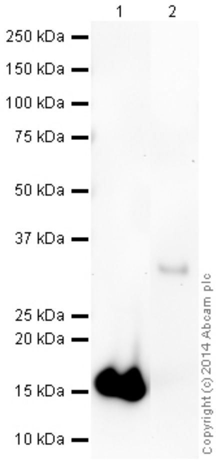

Western blot - Anti-Caspase-3 antibody (ab13847)

All lanes : Anti-Caspase-3 antibody (ab13847) at 1 µg/ml

Lane 1 : Human Caspase 3 (active) Recombinant Protein
Lane 2 : Human Pro Caspase 3 (inactive) Recombinant Protein

Lysates/proteins at 0.1 µg per lane.

Secondary
All lanes : Goat Anti-Rabbit IgG H&L (HRP) (ab97051) at 1/10000 dilution

Developed using the ECL technique.

Performed under reducing conditions.

Predicted band size: 17 kDa
Observed band size: 17,32 kDa

Exposure time: 8 minutes

Caspase 3 exist as inactive proenzymes which undergo proteolytic processing at conserved aspartic residues to produce large

(17kDa) and small (12kDa) subunits. These subunits dimerize to form the active enzyme. ab13847 specifically detects the large active subunit (17kDa) and the inactive pro Caspase 3 (32 kDa).

This blot was produced using a 4-12% Bis-tris gel under the MES buffer system. The gel was run at 200V for 35 minutes before being transferred onto a Nitrocellulose membrane at 30V for 70 minutes. The membrane was then blocked for an hour using 5% Bovine Serum Albumin before being incubated with ab13847 overnight at 4°C. Antibody binding was detected using an anti-rabbit antibody conjugated to HRP, and visualised using ECL development solution.

Secondary antibody - Goat Anti-Rabbit IgG H&L (HRP) (ab97051) secondary antibody

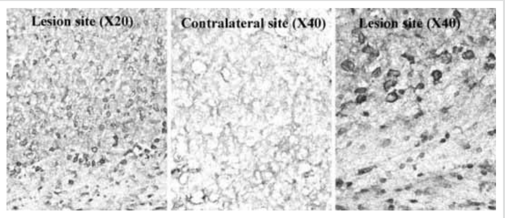

Immunohistochemistry (Frozen sections) - Anti-Caspase-3 antibody (ab13847)

This image is courtesy of an abreview submitted by Sophie Pezet, ESPCI, France

ab13847 was used in IHC of frozen sections from a rat brain with a kainite lesion. The non lesionned contralateral site serves as a negative control. The sections were fixed with paraformaldehyde. The tissue was perfused with 4% PFA and embedded in OCT compound and cut on the cryostat. The primary antibody was incubated for 12 hours at a dilution of 1/300. A biotin labelled secondary antibody was used at a dilution of 1/300.

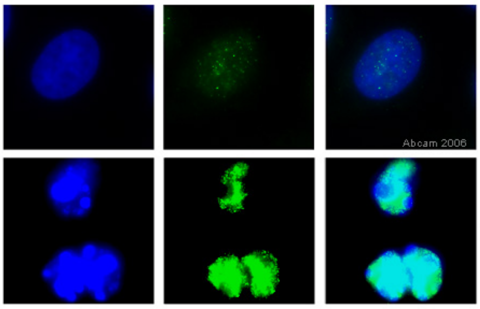

Immunocytochemistry/ Immunofluorescence - Anti-Caspase-3 antibody (ab13847)

This image is courtesy of Roberto Giambruno, Marilena Ciciarello and Patrizia Lavia

HeLa cells were fixed for 10 minutes at room temperature in 3.7% PFA and permeabilised in 0.1% Triton X-100/PBS then incubated with ab13847 (5µg/ml) for 1 hour at room temperature. The top panel shows control cells treated with DMSO. The bottom panel shows HeLa cells treated with 1 mM staurosporine (ab146588) for 4 hours to induce caspase-3 activation. ab13847 staining is shown in green and counterstaining with DAPI is shown in blue. 100x magnification.

The image shows the staining with ab13847 is very faint in the untreated control cultures, but very bright after activation of capsase-3 by treatment with the staurosporine . (N.B. in these cultures the nuclei are apoptotic).

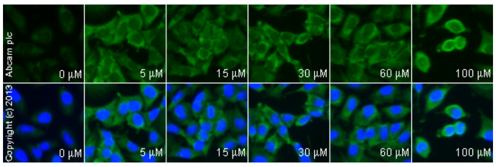

Immunocytochemistry/ Immunofluorescence - Anti-Caspase-3 antibody (ab13847)

ab13847 staining caspase 3 in A549 cells treated with quercetin (ab120247), by ICC/IF. Increase in caspase 3 expression correlates with increased concentration of quercetin, as described in literature.
The cells were incubated at 37°C for 6h in media containing different concentrations of ab120247 (quercetin) in DMSO, fixed with 100% methanol for 5 minutes at -20°C and blocked with PBS containing 10% goat serum, 0.3 M glycine, 1% BSA and 0.1% tween for 2h at room temperature. Staining of the treated cells with ab13847 (1 µg/ml) was performed overnight at 4°C in PBS containing 1% BSA and 0.1% tween. Goat Anti-Rabbit IgG H&L (DyLight® 488) preadsorbed (ab96899) at 1/250 dilution was used as the secondary antibody. Nuclei were counterstained with DAPI and are shown in blue.

Please note: All products are "FOR RESEARCH USE ONLY AND ARE NOT INTENDED FOR DIAGNOSTIC OR THERAPEUTIC USE"

Our Abpromise to you: Quality guaranteed and expert technical support

- Replacement or refund for products not performing as stated on the datasheet
- Valid for 12 months from date of delivery
- Response to your inquiry within 24 hours

- We provide support in Chinese, English, French, German, Japanese and Spanish
- Extensive multi-media technical resources to help you
- We investigate all quality concerns to ensure our products perform to the highest standards

If the product does not perform as described on this datasheet, we will offer a refund or replacement. For full details of the Abpromise, please visit http://www.abcam.com/abpromise or contact our technical team.

Terms and conditions

- Guarantee only valid for products bought direct from Abcam or one of our authorized distributors
